# The Assessment of Attitudes of Medical Doctors towards Psychiatric Patients—A Cross-Sectional Online Survey in Poland

**DOI:** 10.3390/ijerph18126419

**Published:** 2021-06-13

**Authors:** Mateusz Babicki, Kamila Kotowicz, Agnieszka Mastalerz-Migas

**Affiliations:** 1 Department of Family Medicine, Wroclaw Medical University, 51-141 Wroclaw, Poland; agnieszka.mastalerz-migas@umed.wroc.pl; 2 Department and Clinic of Psychiatry, Wroclaw Medical University, 50-367 Wroclaw, Poland; kamila.kotowicz@gmail.com

**Keywords:** stigmatization, psychiatric illnesses, social discrimination, mental health

## Abstract

(1) Introduction: Stigmatization is a multi-level process leading to depreciation of particular social groups. It is particularly visible among people suffering from mental illnesses. Patient stigmatization is a serious problem in psychiatric care; thus, a reliable assessment of its level is important in the context of effective medical interventions. The aim of this paper is to assess the level of stigmatization of psychiatric patients among doctors. (2) Methods: An online, quantitative, CAWI (Computer Assisted Web Interview) study was conducted in the form of an anonymous, voluntary survey addressed to doctors working in Poland. The questionnaire questions included a socio-geographic assessment and questions assessing the level of stigmatization. A standardized psychometric tool, the MICA-4 Scale for doctors, was also used. The results obtained were compared with the evaluation of the existing reports on stigmatization among Polish society. (3) Results: 501 doctors of various specialties and at various stages of career participated in the study. Most of the respondents were women (75%). The average score of MICA-4 obtained by the respondents was 40.26 (minimum 17; maximum 67; SD 8.93). The women’s score was lower than the men’s (*p* = 0.034). (4) Conclusions: Stigmatization of psychiatric patients is a common phenomenon among doctors. The type of performed work and career stage has an impact on the perception of psychiatric patients. Specialists scored highest in the MICA-4 Scale, similarly to physicians of surgical fields. Due to the prevalence of the phenomenon of stigmatization, especially among people who are meant to provide patients with help, there is an urgent need to implement anti-stigma programs.

## 1. Introduction

Stigmatization is a multi-level process leading to depreciation of particular social groups. It is particularly visible among people suffering from mental illnesses. The grounds of stigmatization should be seen, i.e., in a low level of knowledge of the society concerning mental disorders, the population bias towards mentally ill people and their overt discrimination. All these factors are part of the definition of stigmatization, which is a relatively common phenomenon both in the general population and among health care workers, which may result in avoiding seeking help by the people affected by them [1,2,3]. Moreover, stigmatization, especially by relatives and medical staff, has a significant impact on the patient, lowering their hope for recovery, self-esteem and adherence to treatment recommendations [4]. Patients frequently needing specialist consultations avoid them, as they are afraid of the diagnosis of a mental illness and the associated social stigma of mental illness, which is currently considered one of the most socially excluded stigmata [4]. This results in a significant delay in the implementation of appropriate treatment, worsening the prognosis of the illness, the possibility of normal social functioning and quality of life [5,6,7,8,9,10,11,12,13]. These individuals undergo a process of self-stigmatization, which is called a “second illness” leading to lowering of self-esteem and is a major obstacle to their ability to return to their social and family roles and it is also considered an independent predictive factor in the recovery process [14,15]. Considering the multi-level stages of stigmatization process, there is no universal measure of social stigmatization, and individual studies focus on its selected elements [14,16]. The prevention and treatment of mental disorders as well as the fight against stigmatization is one of the greatest challenges in the field of public health in the 21st century. The reports from studies conducted in Poland so far indicate a significant intensification of the stigmatization of psychiatric patients. Reluctance is most pronounced in the economic aspect and the willingness to maintain closer relationships as well as respect for personal dignity and education [17]. Moreover, the research conducted by B. Wciórka and J. Wciórka shows the fact that Polish society has excluded people with a history of mental illness, preventing them from fulfilling appropriate social roles, in particular taking responsibility for other people [18,19,20]. Previous observations showed no differences in the approach of Polish psychiatrists concerning the general population [21]. This may suggest a low effectiveness of appropriate methods of teaching doctors, especially in creating favorable attitudes towards psychiatric patients. Poland is still at the beginning of the road to build a modern mental health care system. For such a system to come true, its construction must become one of the government’s top priorities. It should be emphasized that ultimately stigmatization and self-stigma harm not only the sick and their relatives, but also lead to the marginalization of a significant part of citizens, impoverish the entire society and perpetuate its members’ fears of mental illness and the loss of control over their own lives. Patient stigmatization is a serious problem in psychiatric care; thus, a reliable assessment of its level is important in the context of effective medical interventions [22]. The presence of stigma against people with mental illness among medical health personnel carries less quality of medical service, neglect of patient’s complaints in medical settings, lack of appropriate doctor-patient relationship, and therefore higher mortality [22]. Due to concerns about the social status of the family, many researchers say that disclosing a mental illness is considered “shameful” [23] Based on world reports, it should be borne in mind that health care workers may not show significant differences then the general population [24]. Therefore, it is extremely important to constantly monitor the level of stigma among health care workers, especially doctors other than psychiatrists, to assess the level of stigmatization of psychiatric diseases and, if necessary, implement appropriate programs. The aim of the paper is to assess the level of stigmatization of mental disorders among Polish doctors. The following hypotheses were proposed: (1) Stigmatization is a very common phenomenon among Polish doctors. (2) Women have a higher degree of tolerance than men. (3) The doctors with their own experience with a psychologist, psychiatrist or psychotherapist have a lower level of stigmatization. (4).

## 2. Materials and Methods

### 2.1. Methods

The survey was conducted using a proprietary questionnaire distributed online through a social network and a mailing database. The questionnaire was addressed to professionally active doctors of all specialties, not only mental health specialists living and working in Poland. The survey was published in groups which can be accessed by doctors only. Membership in the groups is associated with an adequate verification, confirming the professional status of their members. To disseminate the questionnaire, a mailing database (whose membership was constituted by verification of the number of the right to practice—with the consent of the administrator) of one of the organizers of training courses for doctors was also used. 

It was an online, quantitative, CAWI (Computer Assisted Web interview) study in the form of an anonymous, voluntary survey completed by the respondents with the use of a computer/telephone/tablet. The survey was conducted in Polish. Prior to the participation in the survey, the respondents were informed about the methodology and objectives of the study, as well as its estimated duration. Then they gave their informed written consent for the participation in the study. The respondents could withdraw from participation at any stage of completing the questionnaire, without providing reasons. The study was conducted according to the guidelines of the Declaration of Helsinki and approved by the Bioethics Committee of the Wroclaw Medical University, Poland (approval number: KB-119/2021). 

The form was divided into three parts. The first one pertained to the sociodemographic status of the study group. It contained questions concerning sex, age, career stage, seniority as well as respondents’ own experience with a psychiatrist, psychologist or psychotherapist with respect to their own selves or the closest members of their families. Further questions evaluated the attitude of the specialists towards mental disorders. They were based on the questions contained in earlier publications commissioned by CBOS [18,19,20], the approach of psychiatrists to mental disorders prepared by A. Kochański, A. Cechnicki [21] as well as the evaluation of online respondents in Poland [17].

The final part contained a psychometric tool-MICA-4 (Mental Illness: Clinician’s Attitudes Scale). It is a scale used to evaluate the attitude of medical students and workers towards mental disorders. The adaptation was based on a translation and back translation of the scale. MICA-4 is a 16-item scale was prepared on the basis of version 2, developed for students. The questions are based on Likert’s six-point scale: strongly agree, agree, agree to some extent, disagree to some extent, disagree and strongly disagree. An adequate numerical value is assigned to each answer. The respondents can score from 16 to 96 points. The analysis is based on the sum of points obtained by the respondents. The higher the scale value, the higher the level of stigmatization. The whole questionnaire can be divided into five main parts:

1. Views regarding the evaluation of mental health/health/health care-questions number 3, 5, 8, 10, 11, 12, 16.
2. Knowledge regarding mental illnesses-questions number 1, 2, 5, 6.
3. Revealing own mental illness-for questions 4 and 7.
4. Evaluating physical and mental health-questions number 8, 13, 14, 15.
5. Evaluating psychiatric patient care-questions number 9, 11, 14.

Please note that some questions were classified under more than one category. Ten questions on the scale should be evaluated in the opposite way, i.e., the statement “strongly disagree” is awarded with 6 points, whereas “strongly agree” is awarded 1 point. This concerns questions 1, 2, 4, 5, 6, 7, 8, 13, 14, 15 [25]. The overall internal consistency of the scale in this study, based on Cronbach’s alpha, was 0.76, similar to that found in other studies using the original version [15,16,22].

### 2.2. Statistical Analysis

Statistical calculations were performed using the Statistica 13 software by StartSoft (TIBCO, Palo Alto, CA, USA). The analyzed variables are of an ordinal and quantitative nature. The chi-square test was used to determine the relationship between the ordinal variables. Basic descriptive statistics were used for interval variables. The normality of distributions of those variables was assessed using three different statistical tests: the Kolmogorov–Smirnov test, Lilliefors test, and Shapiro–Wilk test at the significance level of *p* = 0.05 The statistical significance of differences between mean values was assessed using the nonparametric U-Mann–Whitney test or the Kruskal–Wallis test. The analysis of covariance (ANCOVA) was performed to investigate the differences in the levels of stigma between the groups after co-varying for potential confounding factors, such as age, sex.

In each case, α = 0.05 was deemed to be statistically significant.

## 3. Results

### 3.1. Participants 

Detailed description of the study group is presented in Table 1. The study involved 501 doctors at various stages of career, representing various specialties. The respondents were doctors: during the postgraduate internships, specialist registrars and physicians holding the title of a specialist. The mean age of the study group was 33.64 years (minimum 24; maximum 82; SD 8, 92). Personal experience with mental illnesses was also assessed. At least 37% of the respondents sought the help of a psychologist at least once, 18%-consulted a therapist and the same percentage sought psychiatric help. In the case of 38% of doctors, a member of their family suffered from psychiatric disorders, i.e., addiction, mood disorders, schizophrenia or anxiety disorders.

### 3.2. Evaluation of Stigmatization of Patients with Mental Disorders

When analyzing particular questions included in the questionnaire, attention should be paid to the 14% of answers confirming that a mental illness is shameful and a reason to hide from others. In this case no statistical difference (*p* = 0.66) was observed between men and women, the type of work performed (*p* = 0.48) or the stage of career (*p* = 0.11). Twenty-five percent of the respondents believe that such persons are not dangerous. Only 1% of the respondents believing that all patients suffering from mental disorders are dangerous. The vast majority believes that it only pertains to certain illnesses and women show more apprehension than men—77% women vs. 64% of men were afraid of such patients. In terms of visual assessment of patients—88% of the respondents see no difference in the appearance of a healthy person and a person suffering from a mental disorder. The judgement is not affected by the type of performed work (*p* = 0.18) nor the stage of professional career (*p* = 0.42). A significant difference can be observed in the distribution of answers among sexes as women proved to be more tolerant (*p* = 0.003)—90% of women and 81% of men see no difference in the appearance. 

Social acceptance as well as everyday life in the society are extremely important for the process of adapting to mental illness as well as its treatment and recovery. Among the respondents, 97% of doctors state that they would have nothing against having a friends suffering from a mental illness, 93% a neighbor, and 88% a co-worker. No significant differences were observed with respect to sex (*p* = 0.846, *p* = 0.112, *p* = 0.410) and career stage (*p* = 0.279, *p* = 0.331, *p* = 0.316).

Ten percent of the respondents would not hire a person suffering from a mental illness. No significant differences were observed among women and men (*p* = 0.468), surgical and non-surgical doctors (*p* = 0.255), specialists and specialist registrars (*p* = 0.259). The exact answers are presented in Table 2.

One hundred percent of the respondents stated that people suffering from depression can have a normal life, 99.6% stated the same with regard to neurosis * and 98%-with regard to schizophrenia. The exact results are presented in Table 3.

**Figure 1:**
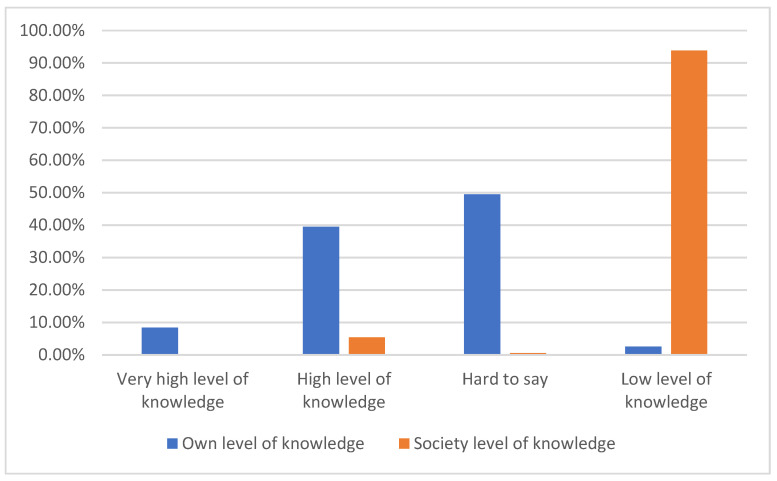
The assess of the level of knowledge about mental disorders.

The respondents were asked to provide their opinion on their own level of knowledge and evaluate the level of knowledge in the society. Both results are presented in Figure 1.

**Figure 2:**
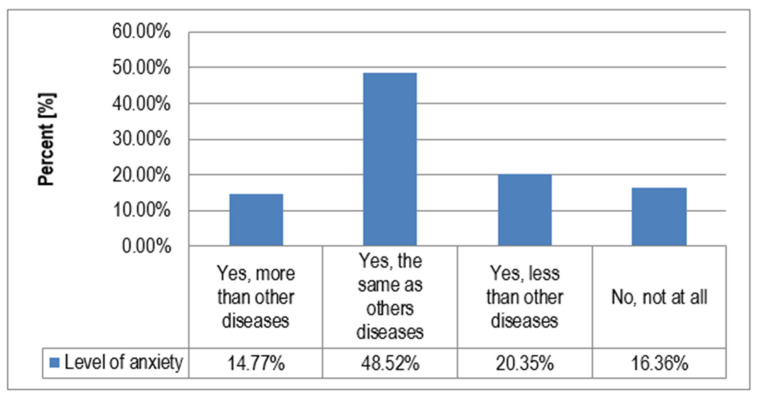
How do you rate your level of knowledge about mental disorders.

The doctors almost unanimously stated that the level of knowledge in our society is low. Only one person stated that it was high and three were unable to give a clear answer. Nearly 84% of the respondents are afraid of developing a mental illness. In the overall evaluation, women show a higher level of fear (*p* = 0.02) than men, 86% vs. 76%, respectively. No significant difference was observed in the answer concerning the intensity of fear: “Yes, more than developing other diseases”. The exact result is presented in Figure 2.

### 3.3. Analysis of Attitudes of Doctors towards SMI (Severe Mental Illness) 

A detailed analysis of the effect of individual factors on MICA-4 score is presented in Table 4. The average result obtained by the respondents was 40.26 (minimum 17; maximum 67; SD 8.93). Women show a more favorable attitude towards mental illness than men (*p* = 0.034). The type of work performed by doctors significantly affects the final result of the MICA-4 scale analysis. Surgical doctors frequently obtained a higher level of stigmatization, compared to interns and non-surgical specialists, *p* < 0.001. The stage of respondents’ career did not have an impact on the final result of MICA-4. (*p* = 0.086). Personal experience with mental illness affects the final results obtained in the overall assessment of the MICA-4 Scale. The attitudes represented by people who have had contact with a psychologist, psychotherapist or a psychiatrist were more favorable than those obtained by the respondents who have not sought specialist help.

The MICA-4 scales can be interpreted at the level of individual questions. Attention should be paid to the high (43%) percentage of the respondents who stated that they only studied psychiatry because they had to. Most of these respondents are doctors performing surgical work—as many as 42% of them admit to that, statistically significantly more often than their colleagues specializing in other fields (*p* < 0.001). Despite the high percentage of respondents who are reluctant to study psychiatry, 11% believe that psychiatry is not a field of science equivalent to other branches of medicine. 

### 3.4. Covariance Analysis

The analysis of covariance (ANCOVA) showed no effect of age on differences in the MICA-4 values with respect to gender (F 1.38; *p* = 0.11), type of work (F 0.76; *p* = 0.77), work experience (F 0.912; *p* = 0.65) as well as one’s own level of knowledge (F 1.07; *p* = 0.33). The gender distribution did not affect the final MICA-4 score in terms of the type of work (F 0.97; *p* = 0.38), seniority (F 1.02; *p* = 0.432) and the assessment of the level of knowledge (F 1.4; *p* = 0.24).

## 4. Discussion

Epidemiological studies conducted in Poland indicate that a significant part of the society suffers from mental disorders which affect people of all ages. The number of people, especially children [26], needing psychiatric help is growing year by year, constituting not only a problem of individuals, but of the entire society.

The published literature shows that the stigmatization of psychiatric patients is a common phenomenon among the Polish society [10,17]. The results obtained from our own research correspond to the general trend of the stigmatization process of mental illnesses. The 2018 study by M. Babicki et al. was used to analyze the similarities/differences. The study is based on an online survey addressed to the general public with the aim of assessing the level of stigmatization of psychiatric illnesses according to online respondents living in Poland. A total of 1309 anonymous, voluntarily recruited respondents were surveyed [17]. Comparing the obtained results of both studies, in Poland, doctors’ evaluation of people suffering from mental diseases is better than that of the general population. The biggest difference is visible in the case of acquainting such people, as only 3% of doctors would mind such an acquaintance, while in the general population the percentage of such responses reached 10.6% (*p* < 0.001). A similar correlation is evident in the evaluation of neighborhood (7% vs. 14% *p* < 0.001) and cooperation with psychiatric patients (12% vs. 18% *p* < 0.001). The reasons for these differences can be seen in many factors. One of them is undoubtedly the above-average level of knowledge about psychiatric diseases by doctors compared to the Polish society. In addition, we assume the hypothesis that in their daily practice doctors more often have contact with people suffering from psychiatric diseases, which has a huge impact on creating anti-stigma attitudes [17]. This was also confirmed in this review, where both people using the services of a psychiatrist, psychologist or psychotherapist showed more favorable attitudes. However, despite the lower percentage of affirmative responses, the proportion of physicians remains unsatisfactory.

When it comes to economic matters concerning employment, in both cases there is a high level of stigmatization—89% of doctors and 90% of Polish society would not employ a person suffering from psychiatric illnesses (*p* = 0.601) [17]. The study conducted by Kochański et al. shows that only 10% of psychiatrists believe that past mental illness does not limit the possibility of gainful employment. Additionally, 33.1% of them would not entrust a task that requires teamwork [21]. This fact may be due to the fear of instability of the mental ability of patients and its effect on the ability to conscientiously perform their duties. In both cases, the economic aspect of stigmatization is very much emphasized. The same conclusions can be drawn from global research from China, where 32.0% of doctors would not employ a person with depression, 44.8% would not employ a person suffering from schizophrenia and 29.9% would not employ a person suffering from anxiety disorders [27]. The lack of willingness by doctors to employ mentally ill people may result from the fact that poor mental health may lead to changes in behavior and thinking, and also increases the risk of burnout, directly translating into the effectiveness of the work performed. In addition, mental diseases may pre-dispose the development of physical diseases such as hypertension and diabetes, which may directly affect the company’s finances resulting from the absenteeism of employees. In addition, it has been shown that service providers are characterized by greater pessimism in the context of full recovery, which is also perceived as a source of stigma and stigma [28].

Fourteen percent (14%) of Polish doctors believe that mental illnesses are a cause for shame and hiding from others, while in the general population this percentage is 19% (*p* = 0.021). In a survey conducted among Polish psychiatrists, as many as 95% of them consider mental illnesses as diseases that are hidden from other people. Shame and non-disclosure of mental illness is a sort of prelude to the initiation of the stigmatization process, and such high scores among doctors may result from the awareness of low social tolerance for mental disorders and the perception of mental illness as one of the strongest stigmata in the modern world [21]. There is no clear consensus in the world literature on the level of stigmatization of psychiatric patients by medical personnel. Some reports contradict the results of this study and health care professionals have shown a greater number of stigmatizing attitudes than the general population [29,30,31]. An in-depth analysis should take into account the cultural conditions of a region, the degree of occupational burnout and the type of analyzed psychiatric illness. However, such reports are not optimistic; on the contrary, they confirm the belief that both the general population and health care workers share a common stigmatizing attitude towards patients suffering from psychiatric disorders [32], which may increase patients’ reluctance to seek medical attention when needed; thus, delaying the implementation of appropriate treatment methods and the return of a person to normal functioning in the family and society [4,5,6,7,8,9,10,11,12,13]. 

Worldwide reports from Tunisia and Latin American countries (Bolivia, Brazil, Chile, Cuba) indicate that Polish doctors reach a high threshold of stigmatization of patients, scoring 40.26 points on the MICA-4 scale compared to 36.3 in South American countries and 28.4 in Tunisia [33,34]. However, a higher level of stigmatization has been found in many other studies using the MICA-4 scale, i.e., in Saudi Arabia, where the average score fluctuated around 45.75 points [35], China (51.69 pts.) [27] and Nigeria [36]. Significant differences between individual countries may result from many aspects, including the distribution of gender, age and direct work with patients suffering from mental disorders among respondents, which significantly affect the level of stigmatization. So are the cultural conditions. It has been proven that members of Asian cultures may display greater stigma than Westerners. The reasons for this phenomenon are seen in the limitation of the possibility of fulfilling appropriate social roles, which are central cultural values. This causes them fear and anxiety about mental illness [27,37]. 

A significant impact on the perception of people with mental illnesses has one’s own experience of the disease. In both our own study and literature there is a direct correlation between the use of psychiatric/psychological or psychotherapeutic services and the assessed level of stigmatization. This phenomenon can be explained by the contact hypothesis, i.e., development of more positive attitude and a lower degree of stigmatization as a result of increased personal and professional interaction with psychiatric disorders [38,39,40,41]. It has also been confirmed in many studies that anti-stigmatization activities achieve much better results by adopting the strategy of direct contact rather than education [27]. It should be noted here that a psychotherapist may also be a psychologist, but with a completed special course giving him the right to conduct psychotherapy. In this study, the division was made based on the purpose of the person visiting a specialist.

The authors are aware of a methodological limitation of this study, which is the method of data collection with the use of the Internet that narrowed the study group to Internet users, mainly social media users. Another methodological limitation of this study is the lack of possibility to assess both the number of questionnaires that were not completed at any stage of the study and the number of individuals reached by the survey. However, current studies show that online surveys are a recognized research method, which enables reaching a wide group of recipients. Due to full anonymity and safety, the obtained results are credible. It should also be noted that the group presented in this study is not representative of Polish doctors. Official epidemiological data show that men constitute 42% of professionally active male doctors, while in the authors’ original study this percentage was 25% of the respondents [42]. This, given that women are much more empathic than men [43]. Similarly, the average age of the participants in this study could have affected the final result of the study, which was 33.6 years. According to the statistics from 2020, the average age of doctors in Poland was 49.5 years [44]. Considering that younger doctors show more favorable attitudes, it should be borne in mind that the results may be underestimated. An additional limitation of this paper are disproportions between the groups regarding the career stage and the type of work performed. However, there are currently no public data on the exact number of specialist doctors and registrars in Poland, distinguishing between particular professions, which makes it impossible to assess the distribution of the group in relation to real values. The data published by the Supreme Medical Chamber refer only to the number of specialists in a given field, without any distinction per person, i.e., one doctor with a greater number of specializations is included in each of them separately [42]. In our research only 47.9% of respondents described their level of knowledge about mental disorders as high or very high. This could have had a major impact on final result of the research due to that hat the current scientific reports indicate that the intensity of stigmatization is closely related to the level of knowledge about psychiatric diseases [44].

Summing up, due to the high level of stigmatization of psychiatric patients, there is an immediate need to look for solutions to counteract this phenomenon. Therefore, the power of mass media, of which the contribution to the creation of social attitudes is invaluable, should also be noted. Such a phenomenon opens up the possibilities of using the media to spread knowledge, promote social tolerance and, thus, it may be a powerful tool in the fight against stigmatization pertaining not only psychiatric patients, but all aspects of life. It is extremely important to implement appropriate anti-stigma programs that will have proven their efficacy in reducing the severity of the phenomenon [45,46]. A study conducted on students from medical universities in Poland indicates the emergence of stigmatization of psychiatry and psychiatric patients at the stage of education, before starting their professional career. Moreover, the completed course of psychiatry has not been shown to reduce the phenomenon of stigma [47]. This may suggest that the main cause of stigmatization of psychiatric patients by doctors is the ineffective teaching of psychiatry during studies, and modifications of the curriculum and teaching method should be considered, increasing the emphasis on direct contact with the patient. Currently, the modernization of the psychiatric care system is underway in Poland, moving it towards social psychiatry. Mental health centers are established, offering numerous innovative interventions and support programs. It also makes an ideal support program for studying psychiatry [48]. Therefore, we hope that the new teaching model will reduce the level of stigma among students, which in the future will translate into their daily work with psychiatric patients. The authors see the necessity of further exploring the topic, especially in a few years when adepts after an innovative course in psychiatry appear on the labor market.

## 5. Conclusions

Stigmatization of psychiatric patients is a common phenomenon—not only in the general population, but also among doctors. The type of performed work has an impact on the perception of psychiatric patients. Specialists scored highest in the MICA-4 Scale, similarly to physicians of surgical fields. Women have a higher degree of tolerance than men. Own experience with a psychologist, psychiatrist or psychotherapist has a significant impact destigmatizing the attitudes of the respondents. Due to the prevalence of the phenomenon of stigmatization, especially among people who are meant to provide patients with help, there is an urgent need to implement anti-stigma programs.

## Figures and Tables

**Table 1:** Characteristics of the study group.

Variable		*n* = 501 *n* (%) M ± SD
Sex	Male	122 (24.4)
	Female	379 (75.6)
Age		33.64 ± 8.92
Type of work	Surgical	90 (17.7)
	Nonsurgical	365 (73.1)
	Intern	4.6 (9.2)
Career stage	Specialist	152 (30.3)
	Specialist registrar	279 (55.7)
	Has not begun specialist training	24 (4.8)
	Intern	4.6 (9.2)
Place of residence	City of more than 500,000 residents	252 (50.3)
	City of 100,000–500,000 residents	108 (21.6)
	Town of 50,000–100,000 residents	42 (8.4)
	Town less than 50,000 residents	65 (13.0)
	Country	34 (6.7)
Experience with psychologist	Yes, on a regular basis	45 (8.9)
	Yes, sometimes	74 (14.8)
	Yes, once	65 (13.0)
	No	317 (63.3)
Experience with psychotherapist	Yes, on a regular basis	63 (12.5)
	Yes, sometimes	50 (10.0)
	Yes, once	27 (5.4)
	No	361 (72.1)
Experience with psychiatrist	Yes, on a regular basis	23 (4.5)
	Yes, sometimes	54 (10.8)
	Yes, once	63 (12.6)
	No	361 (72.1)
Pharmacological treatment	Yes	131 (26.1)
	No	370 (73.9)
Mental illness of a loved one	Yes	192 (38.3)
	No	309 (61.7)
Being a tolerant person	Yes	478 (95.4)
	No	23 (4.6)
Work experience [years]	-	7.63 ± 9.03

**Table 2:** Distribution of answers of respondents assessing the level of stigmatization.

Question	Yes [%]		No [%]
Would you have anything against a person suffering from a mental illness being your neighbor?	7%		93%
Would you have anything against a person suffering from a mental illness being your co-worker?	12%		88%
Would you have anything against being friends with a person suffering from a mental illness?	3%		97%
If you were an employer, would you hire a person who is seeing a psychiatrist?	10%		90%
Are mental disorders shameful and should be hidden from others?	14%		86%
Have you ever encountered a social campaign concerning people with mental illnesses?	55%		45%
Do you think that people suffering from a mental illness are dangerous?	Yes, patients suffering from all mental illnesses are dangerous	Yes, patients with some mental illnesses are dangerous	25%
	1%	74%	
Does the appearance of a person suffering from a mental illness differ from that of other people?	0.41%	88%	11%

**Table 3:** Distribution of answers regarding the ability to function in the society.

Question	Yes, Always	Yes, After the Completion of Treatment	Yes, during Treatment	No, Never
Do you believe that people suffering from depression can function normally in the society, for example go to work, take care of children, shop?	53%	38%	9%	0%
Do you believe that people suffering from neurosis * can function normally in the society, for example go to work, take care of children, shop?	45%	7%	48%	0.41%
Do you believe that people suffering from schizophrenia can function normally in the society, for example go to work, take care of children, shop?	15%	14%	69%	2%

Neurosis * such as anxiety disorders, hysteria, obsessive–compulsive disorders, impulse control disorder.

**Table 4:** A detailed analysis of the effect of individual factors on MICA-4 score.

Variable		Mean ± SD	*p*
Sex *	Male	41.66 ± 8.82	0.034
	Female	39.82 ± 8.94	
Type of work #	Surgical	44.18 ± 8.51	<0.001
	Nonsurgical	39.75 ± 8.86	
	Intern	36.51 ± 7.82	
Career stage #	Specialist	41.51 ± 8.81	0.086
	Specialist registrar	40.28 ± 9.02	
	Has not begun specialist training	39.29 ± 8.87	
	Intern	36.52 ± 7.74	
Place of residence #	City of more than 500,000 residents	39.81 ± 8.74	0.048
	City of 100,000–500,000 residents	39.03 ± 8.78	
	Town of 50,000–100,000 residents	42.57 ± 9.91	
	Town less than 50,000 residents	42.48 ± 8.51	
	Country	40.41 ± 9.71	
Experience with psychologist #	Yes, on a regular basis	37.04 ± 8.50	<0.001
	Yes, sometimes	38.97 ± 8.62	
	Yes, once	39.15 ± 9.83	
	No	41.29 ± 8.74	
Experience with psychotherapist #	Yes, on a regular basis	36.41 ± 7.89	<0.001
	Yes, sometimes	37.62 ± 8.09	
	Yes, once	41.29 ± 5.58	
	No	41.22 ± 9.19	
Experiance with psychiatrist #	Yes, on a regular basis	34.69 ± 8.31	<0.001
	Yes, sometimes	38.11 ± 7.94	
	Yes, once	38.95 ± 7.62	
	No	41.16 ± 9.14	
Pharmacological treatment *	Yes	37.08 ± 8.21	<0.001
	No	41.14 ± 9.03	
Mental illness of a loved one *	Yes	39.22 ± 8.94	0.025
	No	40.91 ± 8.87	
Being a tolerant person *	Yes	39.95 ± 8.82	<0.001
	No	46.83 ± 8.95	
Anxiety of developing a mental illness #	Yes, more than other diseases	43.51 ± 9.32	0.024
	Yes, the same as other diseases	38.58 ± 8.40	
	Yes, less than other diseases	41.21 ± 9.30	
	No, not at all	41.13 ± 8.73	
Own level of knowledge #	Very high	34.52 ± 9.69	<0.001
	High	37.82 ± 8.49	
	Hard to say	42.79 ± 7.99	
	Low	47.69 ± 10.11	
	-	r	*p*
Work experience	-	0.085	0.051
Age	-	0.103	0.021

* U-Mann–Whitney test. # Kruskal–Wallis test.

## Data Availability

The data presented in this study are available on request from the corresponding author.
